# αO-Conotoxin GeXIVA[1,2] Reduced Neuropathic Pain and Changed Gene Expression in Chronic Oxaliplatin-Induced Neuropathy Mice Model

**DOI:** 10.3390/md22010049

**Published:** 2024-01-19

**Authors:** Huanbai Wang, Xiaodan Li, Yamin Qiao, Meiting Wang, Wen Wang, J. Michael McIntosh, Dongting Zhangsun, Sulan Luo

**Affiliations:** 1 Key Laboratory of Tropical Biological Resources of Ministry of Education, Hainan University, Haikou 570228, China; hbwang93@163.com (H.W.); lixiaodan816@163.com (X.L.); qiao_yamin2020@163.com (Y.Q.); wenwangcn@126.com (W.W.); 2 Guangxi Key Laboratory of Special Biomedicine, School of Medicine, Guangxi University, Nanning 530004, China; wmttymt@163.com; 3 Department of Biology and Department of Psychiatry, University of Utah, Salt Lake City, UT 84112, USA; mcintosh.mike@gmail.com; 4 George E. Wahlen Veterans Affairs Medical Center, Salt Lake City, UT 84108, USA

**Keywords:** α9α10 nAChR, αO-conotoxin GeXIVA[1,2], CIPN, neuropathic pain, oxaliplatin, RNA sequencing

## Abstract

Chemotherapy-induced peripheral neuropathy (CIPN) is a dose-limiting painful neuropathy that occurs commonly during cancer management, which often leads to the discontinuation of medication. Previous studies suggest that the α9α10 nicotinic acetylcholine receptor (nAChR)-specific antagonist αO-conotoxin GeXIVA[1,2] is effective in CIPN models; however, the related mechanisms remain unclear. Here, we analyzed the preventive effect of GeXIVA[1,2] on neuropathic pain in the long-term oxaliplatin injection-induced CIPN model. At the end of treatment, lumbar (L4-L6) spinal cord was extracted, and RNA sequencing and bioinformatic analysis were performed to investigate the potential genes and pathways related to CIPN and GeXIVA[1,2]. GeXIVA[1,2] inhibited the development of mechanical allodynia induced by chronic oxaliplatin treatment. Repeated injections of GeXIVA[1,2] for 3 weeks had no effect on the mice’s normal pain threshold or locomotor activity and anxiety-like behavior, as evaluated in the open field test (OFT) and elevated plus maze (EPM). Our RNA sequencing results identified 209 differentially expressed genes (DEGs) in the CIPN model, and simultaneously injecting GeXIVA[1,2] with oxaliplatin altered 53 of the identified DEGs. These reverted genes were significantly enriched in immune-related pathways represented by the cytokine–cytokine receptor interaction pathway. Our findings suggest that GeXIVA[1,2] could be a potential therapeutic compound for chronic oxaliplatin-induced CIPN management.

## 1. Introduction

Cancer is a severe threat to human health that affects people all over the world. Chemotherapy is widely used in treating various types of cancer among the currently accessible cancer therapies [1]. Multiple anti-cancer drugs can induce CIPN, which often leads to the premature discontinuation of cancer treatment owing to accompanying neuropathic pain. Oxaliplatin is a third-generation platinum drug used for the treatment of multiple types of cancer. The prevalence of pain associated with acute oxaliplatin treatment was reported as 55.6%, and the prevalence of chronic neuropathic pain in platin-induced peripheral neuropathy was 49.2% [2]. However, the currently used analgesics, like morphine, have issues such as a lack of efficacy and side effects, including respiratory depression and addiction. [3]. To date, there is no approved preventive therapy for CIPN [4].

α9α10 nAChR was recently verified as a promising target for neuropathic pain treatment. Specific antagonists targeting α9α10 nAChR represented by several conotoxins alleviate and even promote the recovery of neuropathy, suggesting a unique disease-modifying effect [5,6,7]. As previously described, the αO-conotoxin GeXIVA[1,2] discovered in our lab is a selective and potent antagonist targeting the α9α10 nAChR, with IC_50_ of 4.61 nM and 20.3 nM for rat and human receptors, respectively [8,9]. GeXIVA[1,2] displayed analgesia in various neuropathic pain models, including rat CIPN models induced by oxaliplatin and paclitaxel. Repeated GeXIVA[1,2] injection relieved mechanical and cold allodynia in an established acute oxaliplatin injection-induced rat CIPN model [10,11].

However, because CIPN is usually a foreseeable outcome during chemotherapy, a preventive GeXIVA[1,2] treatment schedule concomitant with oxaliplatin injection was applied in the present study. To observe the potential neurological influence of GeXIVA[1,2] on mice behaviors, mice received chronic GeXIVA[1,2] 1.73 mg/kg or vehicle control injection for 3 weeks, and OFT and ELPM were performed weekly. RNA sequencing analysis of L4-L6 spinal cord tissue was performed after the cessation of drug administration to identify key regulated genes and pathways by oxaliplatin and GeXIVA[1,2] injections.

## 2. Results

### 2.1. GeXIVA[1,2] Treatment Alleviated Oxaliplatin-Induced Mechanical Allodynia

**Figure 1:**
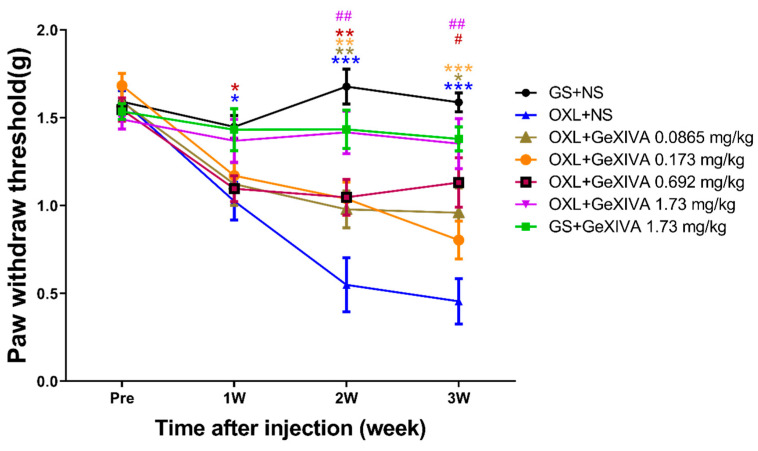
GeXIVA[1,2] treatment reduced chronic oxaliplatin-induced mechanical allodynia. Mechanical PWT evaluated before and once a week after injections. (*n* = 11–12). * = *p* < 0.05, ** = *p* < 0.01, *** = *p* < 0.001 compared with GS + NS control group; # = *p* < 0.05, ## = *p* < 0.01, compared with OXL + NS model group; analyzed by two-way ANOVA followed by Tukey’s post hoc test.

Before and during the treatment procedure, mechanical PWT was measured once per week to evaluate the extent of allodynia (Figure 1). The OXL + NS model group caused mechanical allodynia during treatment, and a significant difference was found in the GS + NS group since week 1 (*p* < 0.05), which peaked at weeks 2–3 (*p* < 0.001). The simultaneous injection of different doses of GeXIVA[1,2] (0.0865—1.73 mg/kg) alleviated the extent of oxaliplatin-induced allodynia. The OXL + GeXIVA[1,2] 1.73 mg/kg group showed no significant difference from the GS + NS control group at all time points (*p* > 0.05). Compared with the OXL + NS model group, the OXL + GeXIVA 0.692 mg/kg group relieved allodynia at week 3 (*p* < 0.05) and the OXL + GeXIVA 1.73 mg/kg group relieved allodynia at week 2 and week 3 (*p* < 0.01). We next compared the PWT of normal mice (GS + NS) and GeXIVA[1,2] 1.73 mg/kg treated mice (GS + GeXIVA); no obvious difference was observed throughout the period (*p* > 0.05).

### 2.2. Chronic GeXIVA[1,2] Injection Did Not Affect Mice Behaviors in OFT and ELPM

**Figure 2:**
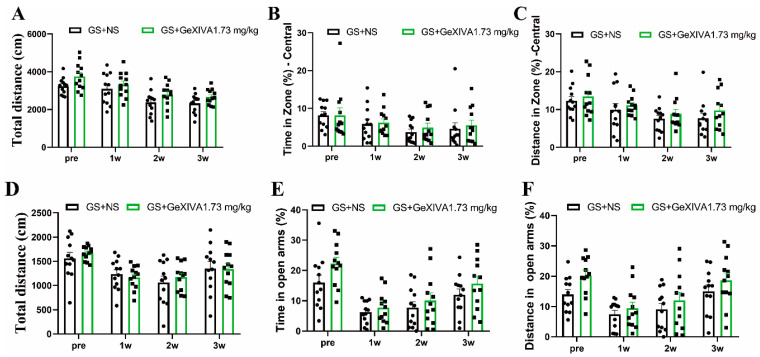
Evaluation of mice behaviors in OFT (**A**–**C**) and ELPM (**D**–**F**) after chronic GeXIVA[1,2] treatment. Data are the mean ± SEM of 12 mice per group. Two-way ANOVA followed by Bonferroni’s post hoc test was performed.

OFT and ELPM were conducted before and weekly during repeated treatment to evaluate the potential effect of chronic GeXIVA[1,2] injection on mice behaviors. In OFT, no significant difference was observed between the GS + NS and GS + GeXIVA groups in terms of total distance (Figure 2A), time in central zone % (Figure 2B), and distance in central zone % (Figure 2C). In ELPM, GS + GeXIVA had no effect in terms of total distance (Figure 2D), time in open arms % (Figure 2E), and distance in open arms % (Figure 2F). These results suggest that chronic s.c injection of GeXIVA[1,2] 1.73 mg/kg/day for 3 weeks had no obvious effect on the mice’s spontaneous activity and anxiety-like behaviors.

### 2.3. Overview of RNA-Seq Data

The quality of RNA-seq data is shown in Table 1. The size of clean data of each sample reached 6.24 G, the map rate reached 94.54%, and the Q20 and Q30 reached 97.37% and 92.89% respectively. The distribution of gene expression levels across samples is shown in Supplementary Figure S1A The correlation heatmap of gene expression levels among samples is shown in Supplementary Figure S1B. The principal component analysis (PCA) results, as illustrated in Supplementary Figure S1C, showed that the four treatment groups were separated.

### 2.4. Differential Gene Expression in the Spinal Cord of CIPN Mice

**Figure 3:**
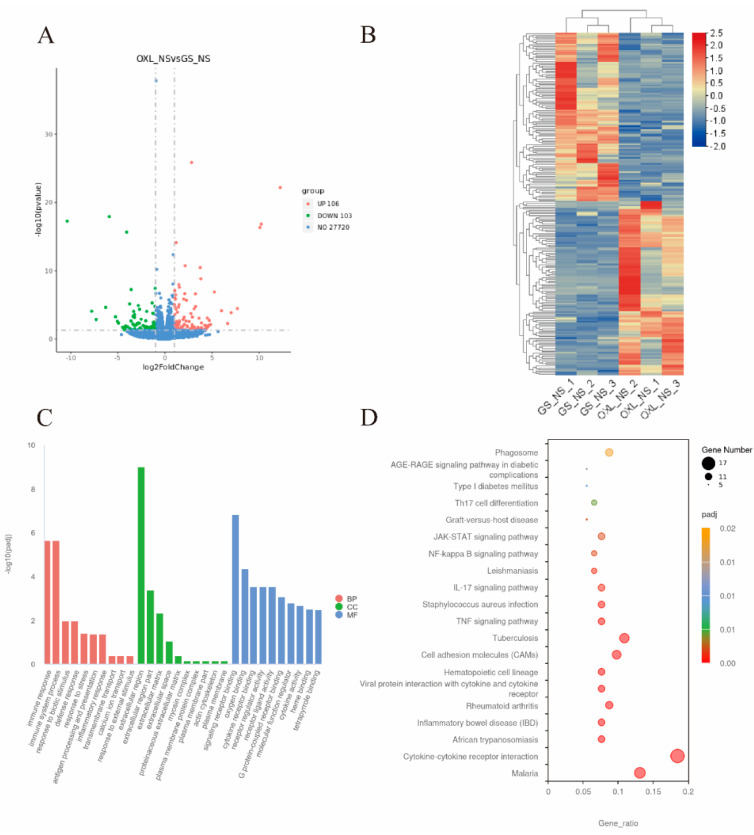
Transcriptional changes of CIPN mice spinal cord. (**A**). DEGs between OXL + NS and GS + NS control group. (**B**) Heatmap of DEGs. (**C**) GO enrichment of DEGs. BP: biological process; CC: cellular component; MF: molecular function. (**D**) KEGG enrichment of DEGs. OXL_NS: i.p. oxaliplatin + s.c. NS group; GS_NS: i.p. GS + s.c. NS group.

RNA sequencing was used to identify DEGs or pathways regulated by chronic oxaliplatin injections. Figure 3A shows the volcano plot of DEGs between the OXL + NS and GS + NS groups, and 106 up- and 102 down-regulated genes were identified. The heatmap of identified DEGs is shown in Figure 3B. Detailed information of the DEGs is shown in the Supplementary File S1.

The GO analysis of DEGs is described in Figure 3C. The enriched biological process was associated with “immune response”, “immune system process”, “response to biotic stimulus”, “defense response”, and “response to stress”; enriched cellular component associated with “extracellular region”, “extracellular region part”, “extracellular matrix”, “extracellular space”; enriched molecular function associated with “signaling receptor binding”, “oxygen binding”, “cytokine receptor binding”, “receptor regulator activity”, and “receptor ligand activity”.

The KEGG analysis, as shown in Figure 3D, identified that DEGs associated with CIPN were mainly enriched in the classifications related to immune or inflammation, like “Cytokine–cytokine receptor interaction”, “Malaria”, “African trypanosomiasis”, “Inflammatory bowel disease (IBD)”, and “Rheumatoid arthritis”.

### 2.5. GeXIVA[1,2] Treatment Altered DEGs Identified in CIPN Model

**Figure 4:**
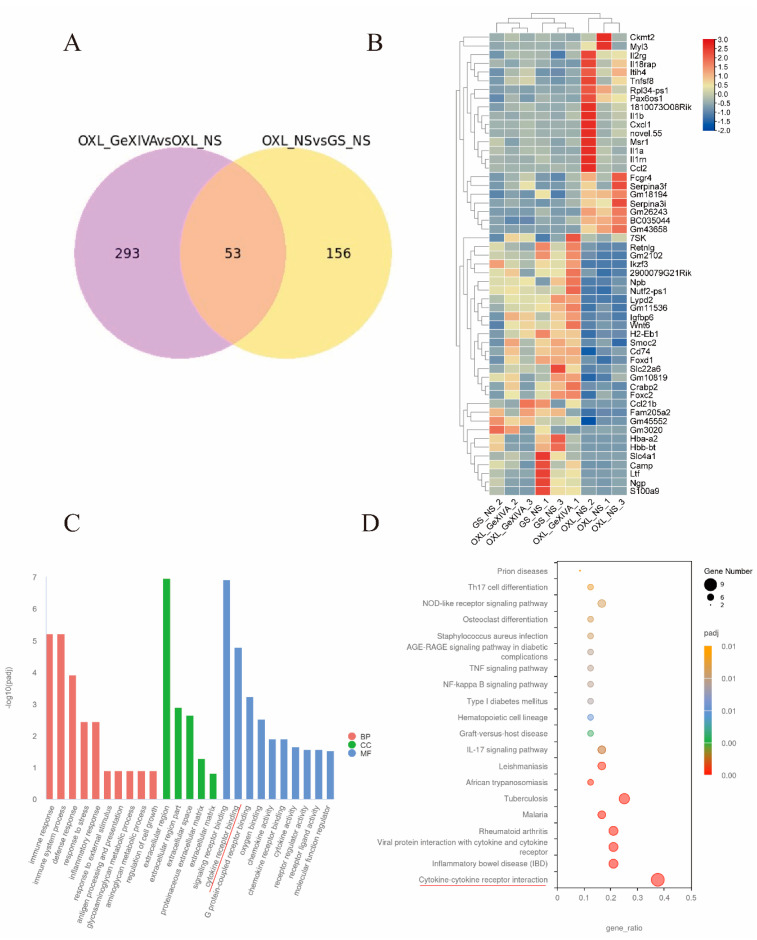
GeXIVA[1,2] altered CIPN-associated DEGs. (**A**). Venn diagram illustrating 53 overlapped DEGs between OXL + NS vs. GS + NS and OXL + GeXIVA vs. OXL + NS comparison. (**B**) Heatmap of 53 overlapped DEGs. (**C**) GO enrichment of 53 overlapped DEGs. BP: biological process; CC: cellular component; MF: molecular function. (**D**) KEGG enrichment of 53 overlapped DEGs. OXL_NS: i.p. oxaliplatin + s.c. NS group; and OXL_GeXIVA: i.p. oxaliplatin + s.c. GeXIVA[1,2] group. The red underlined terms are similar classifications identified in GO and KEGG analysis.

We next compared the changes in gene expression between the OXL + GeXIVA[1,2] treatment group and OXL + NS model group; 346 DEGs were identified, including 233 up- and 113 down-regulated genes (Supplementary File S1). The Venn diagram shown in Figure 4A shows that 53 overlapped DEGs were identified between OXL + NS vs. GS + NS and OXL + GeXIVA vs. OXL + NS comparison (Supplementary File S1). The differential expression level of 53 overlapped genes is shown as a heatmap in Figure 4B, and the OXL + GeXIVA group clustered with the GS + NS group, rather than the OXL + NS group.

The GO analysis results in Figure 4C show that these 53 overlapped DEGs were mainly enriched in the biological process, including “immune response”, “immune system process”, “defense response”, “response to stress”, “inflammatory response”; and cell component including “extracellular region”, “extracellular region part”, “extracellular space”; and molecular function including “signaling receptor binding”, “cytokine receptor binding”, “G protein-coupled receptor binding”, and “oxygen binding”.

**Figure 5:**
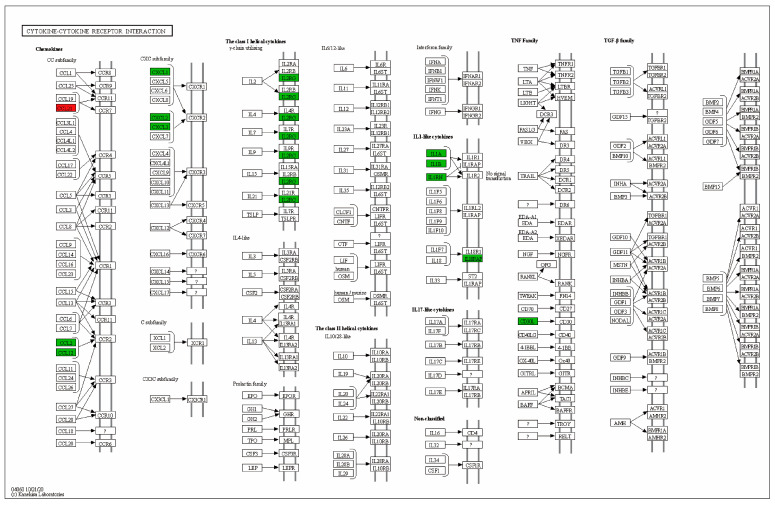
Cytokine–cytokine receptor interaction KEGG pathway. The red and green boxes indicate the up-regulated and down-regulated proteins by GeXIVA[1,2] treatment (OXL + GeXIVA compared with OXL + NS group).

The KEGG analysis results in Figure 4D show that these overlapped DEGs were enriched mainly in “Cytokine–cytokine receptor interaction”, “Inflammatory bowel disease (IBD)”, “Viral protein interaction with cytokine and cytokine receptor”, “Rheumatoid arthritis”, and “Malaria”. The KEGG pathway of the cytokine–cytokine receptor interaction is presented in Figure 5. The proteins regulated by OXL + GeXIVA at the transcriptional level include CCL21 (up-regulated) and CXCL1, CXCL2, CXCL3, CCL2, CCL12, IL2RG, IL1A, IL1B, IL1RN, IL18RAP, and CD30L (down-regulated) compared with the OXL + NS group.

### 2.6. GeXIVA[1,2] Changed Gene Expression in Spinal Cord of Normal Mice

**Figure 6:**
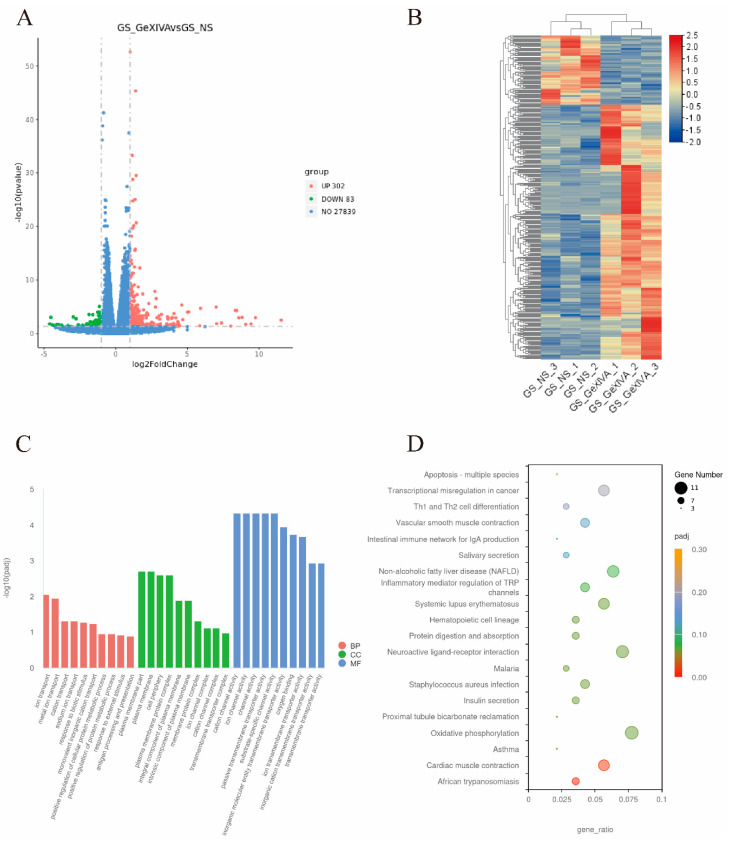
Transcriptional changes in mice spinal cord after chronic GeXIVA[1,2] injections. (**A**). DEGs between GS + GeXIVA and GS + NS control group. (**B**) Heatmap of DEGs. (**C**) GO enrichment of DEGs. BP: biological process; CC: cellular component; and MF: molecular function. (**D**) KEGG enrichment of DEGs. GS_GeXIVA: i.p. GS + s.c. GeXIVA group; and GS_NS: i.p. GS + s.c. NS group.

The RNA sequencing results comparing the gene expression changes between the GS + NS and GS + GeXIVA groups are shown in Figure 6. We found that 385 DEGs were affected by GeXIVA[1,2] injections, including 302 up-regulated and 83 down-regulated genes, as shown in Figure 6A. Detailed information on DEGs is listed in the Supplementary File S1. A heatmap of DEGs is displayed in Figure 6B. The GO analysis result of DEGs is shown in Figure 6C. These genes mainly focused on biological processes, including “ion transport”, “metal ion transport”, “cation transport”, “sodium ion transport”; and cellular component including “plasma membrane part”, “plasma membrane”, “cell periphery”, “plasma membrane protein complex”; and molecular function including “cation channel activity”, “ion channel activity”, “channel activity”, and “passive transmembrane transporter activity”. The KEGG analysis of DEGs mainly indicated enrichment on “African trypanosomiasis” and “Cardiac muscle contraction”.

### 2.7. qRT-PCR Validation

**Figure 7:**
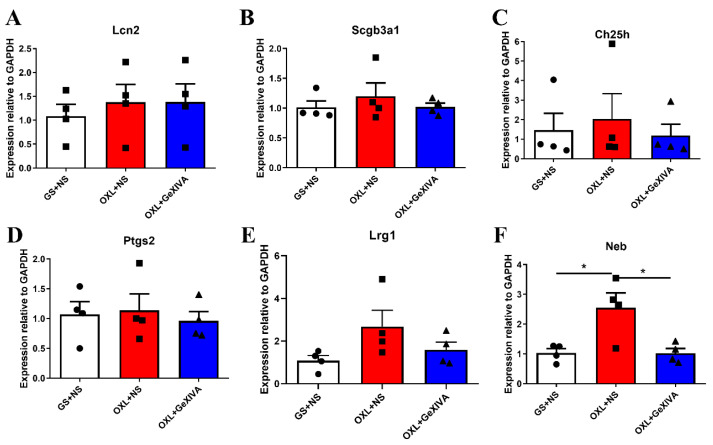
(**A**–**F**) qRT-PCR validation of RNA sequencing results. Data are the mean ± SEM of 4 samples per group. One-way ANOVA followed by Tukey’s post hoc test was performed. GS + NS: i.p. GS + s.c. NS group; OXL + NS: i.p. oxaliplatin + s.c. NS group; and OXL + GeXIVA: i.p. oxaliplatin + s.c. GeXIVA[1,2] group. * indicates *p* < 0.05.

After bioinformatics analysis, six genes were randomly selected for qRT-PCR validation (Figure 7). These selected genes showed a trend of up-regulation in the OXL + NS group compared with the GS + NS control group, or down-regulation in the OXL + GeXIVA group. Among them, Neb was significantly up-regulated in the OXL + NS group and down-regulated in the OXL + GeXIVA group (*p* < 0.05). These results indicate that these selected genes showed a similar trend to the RNA sequencing data.

## 3. Discussion

Oxaliplatin could induce acute neuropathy within hours after a single injection. However, under some circumstances or after chronic injections, the acute phase could be transitioned to a chronic pathological condition, even after chemotherapy treatment terminated (a phenomenon known as coasting) [12]. In previous studies, the α9α10 nAChR antagonists, including RgIA analogs and oligoarginine R8, have been reported to be effective in preventing or alleviating acute and long-term oxaliplatin-induced neuropathy [13,14,15,16]. In the present study, preventive GeXIVA[1,2] injection inhibited chronic oxaliplatin-induced mechanical allodynia and both dosages of GeXIVA[1,2] reduced neuropathic pain to some extent, with the 1.73 mg/kg group inhibiting neuropathic pain at all time points. These results, together with our previous results in an acute oxaliplatin rat model [10], suggest that GeXIVA[1,2] is effective during different states of oxaliplatin-induced neuropathy. Romero et al. reported that RgIA4 prevented the occurrence of chronic oxaliplatin-induced mechanical hyperalgesia and cold allodynia in a broad range of dosages (0.128 μg/kg–80 μg/kg) in rats [17]. Considering that these peptides have a short half-life in vivo, it may be a case that the long-lasting effects of GeXIVA[1,2] are due to disease-modifying effects that are induced by brief low-dose pulses.

We previously reported that acute or chronic GeXIVA[1,2] injection did not affect normal pain perception, grip strength, body weight, and motor performance in rats [10,18]. However, Mohammadi et al. reported that α9 nAChR knockout mice showed increased stress-induced anxiety-like behavior, but not in unstressed animals [19]. Chang et al. reported that α9 nAChR knockout mice displayed impaired postural stability [20]. These results suggest a potential neurological influence when targeting this receptor. In the present study, we continually injected GeXIVA[1,2] 1.73 mg/kg/day in normal mice for 3 weeks, and no significant influence on mechanical pain threshold and mice spontaneous activity and anxiety-like behaviors was discovered, suggesting that GeXIVA[1,2] displayed a good safety profile at the dose that inhibited mechanical allodynia in the CIPN model. In previous RgIA research, the chronic injection of RgIA for 3 weeks showed no influence in the Irwin test (evaluation of autonomic, neurological, and behavioral changes after drug injection) [16]. Wala et al. reported that the non-peptide small-molecule-selective α9α10 nAChR antagonist ZZ1-61c displayed both preventive and therapeutic effects on vincristine-induced neuropathic pain, with no observed side effects in motor coordination and muscle strength [21]. These molecules are both less likely to enter the CNS and selectively block α9α10 nAChR, suggesting that peripherally blocking this receptor in physiological circumstances is relatively safe. However, α9* nAChR may play a vital role during development, and future studies using approaches such as gene knock-down by AAV or chemical genetics in different stages of development could contribute to the understanding of the neurological effect of manipulating α9* nAChR.

The mechanism of CIPN is complicated and involves both the peripheral and central nervous systems. Among the diverse mechanisms, the neuroimmune mechanism plays a vital role in CIPN development. The activation of spinal glia or release of inflammatory factors were reported in bortezomib [22], paclitaxel [23], vincristine [24], and cisplatin-induced CIPN [25]. The up-regulation of pro-inflammatory cytokines and chemokines in the spinal cord has also been suggested to contribute to oxaliplatin-related neuropathic pain [12,26,27]. In the present study, we utilized RNA sequencing analysis to explore changes in gene expression in mice L4–L6 spinal cords after chronic oxaliplatin treatment for the following reasons. Firstly, the spinal cord plays a vital role in pain signal transmission, and the L4–L6 section is commonly used in neuropathic pain related to nerve injury or CIPN studies [28,29]. The transition from acute to chronic pain involves central neuroinflammation mechanisms like glial activation [30], and we used a chronic oxaliplatin-induced CIPN model in this research. Secondly, previous RgIA results showed that the peripherally acting α9α10 nAChR antagonist could affect CNS glial cells [16]. GeXIVA[1,2] and RgIA are both conotoxin-derived short peptides, and both selectively block α9α10 nAChR. These similarities lead us to speculate that GeXIVA[1,2] can act on CNS by peripheral injection. We found that immune-related pathways were altered, suggesting vital roles of cytokines and chemokines in chronic oxaliplatin-induced CIPN.

α9α10 nAChR has been proven to be expressed in immune cells and mediate non-canonical metabotropic pathways that regulate the expression of pro-inflammatory cytokine IL-β [31]. Tsetlin et al. reported that α9α10 nAChR antagonists enhanced the expression of the anti-inflammatory cytokine IL-10 on mouse bone marrow granulocytes upon LPS stimulation [32]. AlSharari et al. reported that the α9α10 nAChR antagonist RgIA was effective in an experimental colitis mice model and reduced the increased level of TNF-α, suggesting α9α10 nAChR as a potential target for inflammatory disorders [33]. Experimental autoimmune encephalomyelitis is a mouse model for multiple sclerosis, and α9/α10 nAChR double knockout reduced inflammatory infiltration to the brain and spinal cord [34]. A brain-controlled immune response pathway was recently described to rely on α9 nAChR. The activation of the corticotropin-releasing hormone (CRH) expressing neurons in the central nucleus of the amygdala (CeA) and the paraventricular nucleus (PVN) could increase the expression of antigen-specific IgG antibodies through the splenic nerve and α9 nAChRs expressed in B cells after immunization [35]. Huynh et al. reported that the preventive effect of α9α10 nAChR antagonist RgIA4 on cold allodynia in an acute oxaliplatin CIPN model relies on CD3+ T-Cells [15]. Richter et al. synthesized an α9α10 nAChR agonist pCN-diEPP and an antagonist mCN-diEPP, and they both attenuated complete Freund’s adjuvant-induced mice inflammatory pain [36]. Our previous pharmacokinetic modeling-based mechanistic assessment suggested that the analgesic effect of GeXIVA[1,2] was associated with the inhibition of endogenous substances [37]. These results suggest that the therapeutic effects of GeXIVA[1,2] in the CIPN model could possibly be attributed to an immune-regulatory mechanism and may involve different types of immune cells.

In the present study, we found that some oxaliplatin-induced DEGs were reverted to control group level by simultaneous GeXIVA[1,2] treatment. These reverted genes are associated with immune-related pathways, represented by the cytokine–cytokine receptor interaction KEGG pathway (Figure 5). The central nervous system neuroimmune mechanism was mainly related to the infiltration of immune cells and hyperactivity of astrocytes or microglia in nerve injury and CIPN models. Cytokines are involved in the immune response, and are released by immune cells, glia, and neurons [12]. During a noxious status such as nerve injury or chronic inflammation, sustained central sensitization leads to altered gene expression and function of spinal dorsal horn neurons and leads to the activation of glia and release of endogenous signals, such as cytokines [38].

In a chronic oxaliplatin-induced rat CIPN model, astrocyte hyperactivity was observed, and the prevention of astrocyte activation reduced oxaliplatin-dependent pain [39,40]. Preventive RgIA treatment alleviated spinal cord astrocyte activation and neuropathic pain induced by oxaliplatin, suggesting a vital role of α9α10 nAChR antagonist in spinal glial activation [16]. A similar effect of RgIA on glial density was also observed in a chronic constriction injury neuropathic pain model [41]. Immune cell infiltration to the spinal cord contributes to CIPN caused by vinca alkaloids and proteasome inhibitors. However, there is a lack of evidence supporting its role in oxaliplatin-induced CIPN [12]. Janes et al. reported that, in a chronic oxaliplatin-induced rat CIPN model, oxaliplatin-induced mechanical hypersensitivity was associated with the hyperactivation of spinal astrocytes, an increased level of pro-inflammatory cytokine, and a decreased level of anti-inflammatory cytokines, but not lymphocytic mobilization, since CD45+/CD3+ T-cell infiltration into the spinal cord was not observed [42]. We did not assess the extent of glia activation after chronic oxaliplatin or GeXIVA[1,2] treatment. However, based on these results, we speculate that the inhibition of overactivated neuron-glial communication may contribute to the observed therapeutic effect of GeXIVA[1,2] against chronic oxaliplatin-induced mechanical allodynia.

As a short peptide, GeXIVA[1,2] is not likely to pass the blood–brain barrier. Moreover, α9α10 nAChR is commonly regarded to not be present in the central nervous system [43]. In this study, although GeXIVA[1,2] injection did not affect normal pain perception or alter spontaneous and anxiety-like behaviors in mice, changes in gene expression were observed in the spinal cord after chronic treatment with GeXIVA[1,2] for 3 weeks. Chronic GeXIVA[1,2] injection for 3 weeks in normal mice changed the expression of 385 genes compared with the control group. These DEGs were significantly enriched in pathways associated with ion channel activity and cardiac muscle contraction, indicating a potential influence on neural activity. In a previous study, the chronic injection of RgIA for 3 weeks increased glial density in the spinal cord and multiple brain areas [16]. These results suggest a secondary central nervous system effect of peripherally injected α9α10 nAChR antagonists; further studies exploring the role of these DEGs could contribute to the understanding of mechanisms related to GeXIVA[1,2] effects.

In summary, we evaluated the effects of GeXIVA[1,2] in a chronic oxaliplatin-induced CIPN model. GeXIVA[1,2] was effective in inhibiting neuropathic pain caused by chronic oxaliplatin, while it did not affect normal pain perception or spontaneous and neurological behaviors in mice. Using RNA sequencing, we explored the transcriptional changes after chronic oxaliplatin and GeXIVA[1,2] injections. These results provide information for the understanding of the role of α9α10 nAChRs in CIPN. GeXIVA[1,2], the α9α10 nAChR-specific antagonist, represents a promising lead for a novel drug for neuropathic pain caused by oxaliplatin.

## 4. Materials and Methods

### 4.1. Animals

Male and female C57BL/6 mice (6 weeks) were obtained from SJA Laboratory Animal Co., Ltd. (Changsha, China). Both male and female mice (50% of each sex) were included in all studies. Animals were kept in groups of 6 in clear plastic cages with food and water supplied ad libitum. The housing room was set with a 12 h light/dark cycle (light on from 8:00 am to 8:00 pm). After arriving at our lab, the animals were allowed 3–5 days to acclimate to the housing and experimental environment; during this phase, the animals were handled once per day for at least 3 days. All the experiments were performed under the guidance of The International Association For The Study Of Pain (IASP) guidelines, and efforts were made to minimize the number and discomfort of animals [44].

### 4.2. Peptide Synthesis

The synthesis and purification of GeXIVA[1,2] were carried out as previously described. In short, the linear peptide with side-chain protection was synthesized using Fmoc chemistry solid-phase methodology, and a two-step oxidation method was used to connect the disulfide bridge [45]. LCMS and UPLC were used to confirm the molecular weight and purity of the compound [8].

### 4.3. Oxaliplatin Treatment and Experimental Schedule

A chronic oxaliplatin model was created by injecting i.p. 3.5 mg/kg/day oxaliplatin (BBI life science) in 5% glucose solution (GS) to mice 5 days a week for 3 weeks (15 injections) [13]. Additionally, mice were injected s.c with different doses of GeXIVA[1,2] or normal saline (NS) vehicle. Two control groups of mice were injected with i.p. GS + s.c. NS and i.p. GS + s.c. GeXIVA[1,2].

### 4.4. Behavioral Tests

#### 4.4.1. Mechanical Allodynia Testing

The mechanical allodynia was evaluated by measuring the mechanical PWT by using the von Frey test method. Briefly, before the test, mice were placed on an elevated platform with a wire netting floor and shrouded by a plexiglass box and were allowed to acclimatize for 30 mins.. Once the mice calmed down, a set of von Frey filaments was used to stimulate the plantar of the right hind limb vertically for 2–5 s. Pain-related behaviors were observed during stimulation; a positive response was defined as unexpected paw withdrawal or licking the hind limb. Then, the filament with fewer grams was used for the next test; otherwise, the filament with more grams was used. When the measurements first crossed, 4 more tests were performed, and the 6 values were analyzed by using the up/down method to calculate the 50% mechanical PWT. The researchers were blinded to the treatment group of the tested animals [46,47].

#### 4.4.2. Open Field Test

Mice were first placed in the test room for 30 min to adapt to the environment before the test began. The OFT apparatus was a square plastic box (40 × 40 × 35 cm); mice were placed at the center of the chamber and allowed to explore for 10 min. The behaviors of mice were recorded and analyzed by using the SMART 3 software (Panlab Harvard Apparatus, Barcelona, Spain) [48].

#### 4.4.3. Elevated Plus Maze Test

The mice were allowed 30 min to adapt to the testing environment before the experiment started. The EPM apparatus was an elevated maze (40 cm height) with two open arms and two closed arms (32 × 8.5 cm each). A mouse was placed in the central area and faced the open arm; 5 min of free exploration was recorded using a camera and analyzed using the SMART 3 software [48].

### 4.5. RNA Sequencing and qRT-PCR

After the last behavior tests, mice were sacrificed by cervical dislocation and the L4–L6 spinal cord tissue was dissected and rinsed in cold PBS solution. The tissue was then put into a freezing tube and flash-frozen in liquid nitrogen, and then stored at −80 °C. Total RNA was extracted using Trizol reagent (Ambion Inc., Austin, TX, USA) according to the manufacturer’s instructions. For RNA sequencing, 4 samples were pooled as 1 biological replicate, and each group included 3 replicates. RNA integrity was assessed using the RNA Nano 6000 Assay Kit of the Bioanalyzer 2100 system. Poly-T oligo-attached magnetic beads were used to purify mRNA from the total RNA. The obtained mRNA was then fragmented in First Strand Synthesis Reaction Buffer. The fragmented mRNA was used as a template to synthesize the first strand of cDNA by using a random hexamer primer and M-MuLV Reverse Transcriptase. The second strain of cDNA was synthesized using dNTPs, DNA Polymerase I, and RNase H. The purified double-strand cDNA was then subjected to terminal repair, 3′ ends adenylation, and connection to the sequencing adapter. Then, an AMPure XP beads system (Beckman Coulter, Beverly, MA, USA) was used to select cDNA with the length preferentially within 370–420 bp. The selected cDNA was then amplified by PCR and purified by AMPure XP. An Agilent Bioanalyzer 2100 system was used for quality assessment. After cluster generation, the library preparations were sequenced on an Illumina NovaSeq 6000 platform at Novogene Co., Ltd., Beijing, China. The sequencing read length was 150 bp paired-end reads. The RNA sequencing raw data have been deposited in the Gene Expression Omnibus—NCBI (https://www.ncbi.nlm.nih.gov/geo/) with the accession number GSE253183 (accessed on 18 January 2024).

For real-time quantitative PCR (qRT-PCR) validation, each sample was considered as 1 biological replicate to extract total RNA. The concentration of total RNA was measured by a Nanodrop Spectrophotometer, and integrity was verified by agarose gel. HiScript^®^ II Q Select RT SuperMix was used for the reverse-transcription of cDNA. ChamQ SYBR qPCR Master Mix (Vazyme Biotech Co., Ltd., Nanjing, China) was used for qRT-PCR in the qTOWER 3 Real-Time PCR Thermal Cycler (Jena Analytical Instruments GmbH, Jena, Germany). The mRNA expression level was calculated using the 2^−ΔΔCt^ equation and presented as the fold-change compared with the GS + NS control group after normalization to *gapdh* mRNA levels. The primers are listed in Table 2.

### 4.6. Bioinformatic Analysis

Bioinformatic analysis was mainly performed using the NovoMagic platform (https://magic.novogene.com/, accessed on 24 December 2023). DESeq2 was used for differential gene expression analysis, and a fold change of ≥2 and *p*-value of <0.05 were set as criteria for screening [49,50]. ClusterProfiler was used for GO and KEGG enrichment analysis [51]. The heat map was generated by using TBtools [52]. The KEGG map was generated by using KEGG Mapper (https://www.genome.jp/kegg/mapper/, accessed on 24 December 2023).

### 4.7. Statistical Analysis

Data were represented as the mean ± SEM and processed by using GraphPad Prism 8. Data were analyzed by one-way or two-way ANOVA with post hoc Tukey’s or Bonferroni’s test. *p* < 0.05 was considered as the standard for a significant difference.

## Figures and Tables

**Table 1:** The summary of the raw RNA sequencing data set.

Sample	Raw_Reads	Clean_Reads	Clean_Bases	Total_Map	Q20	Q30
GS_NS_1	46716272	45655274	6.85 G	44223518 (96.86%)	98.11	94.62
GS_NS_2	44480262	42952690	6.44 G	41313746 (96.18%)	98.02	94.45
GS_NS_3	46572300	43424244	6.51 G	41201614 (94.88%)	97.72	93.79
GS_GeXIVA_1	44574590	42223054	6.33 G	40322767 (95.5%)	97.83	94.1
GS_GeXIVA_2	46047054	44190706	6.63 G	42161490 (95.41%)	97.79	93.97
GS_GeXIVA_3	48388854	46555380	6.98 G	44491735 (95.57%)	98.1	94.7
OXL_GeXIVA_1	52600066	48849060	7.33 G	46288111 (94.76%)	97.85	94.09
OXL_GeXIVA_2	47023926	44871854	6.73 G	42765248 (95.31%)	97.65	93.65
OXL_GeXIVA_3	47806906	47702712	7.16 G	45096434 (94.54%)	97.9	94.27
OXL_NS_1	45368676	43316100	6.5 G	41569301 (95.97%)	97.37	92.89
OXL_NS_2	44891890	43867380	6.58 G	42222855 (96.25%)	97.65	93.49
OXL_NS_3	42498354	41571208	6.24 G	39995953 (96.21%)	97.71	93.7

Summary of RNA sequencing data of 12 samples. Q20 and Q30: Phred quality scores 20 and 30.

**Table 2:** List of primers used for qRT-PCR analysis

Gene Symbol	Full Name	Forward Primer (5′~3′)	Reverse Primer (5′~3′)
Lcn2	Lipocalin 2	TGGCCCTGAGTGTCATGTG	CTCTTGTAGCTCATAGATGGTGC
Scgb3a1	Secretoglobin, family 3A, member 1	ACCACCACCTTTCTAGTGCTC	GGCTTAATGGTAGGCTAGGCA
Ch25h	Cholesterol 25-hydroxylase	TGCTACAACGGTTCGGAGC	AGAAGCCCACGTAAGTGATGAT
Ptgs2	Prostaglandin-endoperoxide synthase 2	TGAGCAACTATTCCAAACCAGC	GCACGTAGTCTTCGATCACTATC
Lrg1	Leucine-rich alpha-2-glycoprotein 1	TTGGCAGCATCAAGGAAGC	CAGATGGACAGTGTCGGCA
Neb	Nebulin	CTCCTGCCGACATGCTGAG	CAGCTCGATGTTCATTGCGTC
Gapdh	Glyceraldehyde-3-phosphate dehydrogenase	AGGTCGGTGTGAACGGATTTG	TGTAGACCATGTAGTTGAGGTCA

## Data Availability

The data presented in this study are available on request from the corresponding author.

## Supplementary Materials

The following supporting information can be downloaded at: https://www.mdpi.com/article/10.3390/md22010049/s1, Figure S1: RNA-sequencing results. (A) The distribution of gene expression levels in each sample. (B) Heatmap of pearson correlation between samples. (C) Principal component analysis of all samples.

## References

[1] Chamberlain M.C. (2010). Neurotoxicity of cancer treatment. Curr. Oncol. Rep..

[2] Brozou V., Vadalouca A., Zis P. (2018). Pain in Platin-Induced Neuropathies: A Systematic Review and Meta-Analysis. Pain Ther..

[3] Martínez-Navarro M., Maldonado R., Baños J.E. (2019). Why mu-opioid agonists have less analgesic efficacy in neuropathic pain?. Eur. J. Pain.

[4] Kim J.H., Dougherty P.M., Abdi S. (2015). Basic science and clinical management of painful and non-painful chemotherapy-related neuropathy. Gynecol. Oncol..

[5] Hone A.J., Servent D., McIntosh J.M. (2018). α9-containing nicotinic acetylcholine receptors and the modulation of pain. Br. J. Pharmacol..

[6] Li X., Tae H.S., Chu Y., Jiang T., Adams D.J., Yu R. (2021). Medicinal chemistry, pharmacology, and therapeutic potential of α-conotoxins antagonizing the α9α10 nicotinic acetylcholine receptor. Pharmacol. Ther..

[7] Hone A.J., McIntosh J.M. (2023). Nicotinic acetylcholine receptors: Therapeutic targets for novel ligands to treat pain and inflammation. Pharmacol. Res..

[8] Luo S., Zhangsun D., Harvey P.J., Kaas Q., Wu Y., Zhu X., Hu Y., Li X., Tsetlin V.I., Christensen S. (2015). Cloning, synthesis, and characterization of αO-conotoxin GeXIVA, a potent α9α10 nicotinic acetylcholine receptor antagonist. Proc. Natl. Acad. Sci. USA.

[9] Zhangsun D., Zhu X., Kaas Q., Wu Y., Craik D.J., McIntosh J.M., Luo S. (2017). αO-Conotoxin GeXIVA disulfide bond isomers exhibit differential sensitivity for various nicotinic acetylcholine receptors but retain potency and selectivity for the human α9α10 subtype. Neuropharmacology.

[10] Wang H., Li X., Zhangsun D., Yu G., Su R., Luo S. (2019). The α9α10 Nicotinic Acetylcholine Receptor Antagonist αO-Conotoxin GeXIVA[1,2] Alleviates and Reverses Chemotherapy-Induced Neuropathic Pain. Mar. Drugs.

[11] Li Z., Han X., Hong X., Li X., Gao J., Zhang H., Zheng A. (2021). Lyophilization Serves as an Effective Strategy for Drug Development of the α9α10 Nicotinic Acetylcholine Receptor Antagonist α-Conotoxin GeXIVA[1,2]. Mar. Drugs.

[12] Fumagalli G., Monza L., Cavaletti G., Rigolio R., Meregalli C. (2020). Neuroinflammatory Process Involved in Different Preclinical Models of Chemotherapy-Induced Peripheral Neuropathy. Front. Immunol..

[13] Christensen S.B., Hone A.J., Roux I., Kniazeff J., Pin J.P., Upert G., Servent D., Glowatzki E., McIntosh J.M. (2017). RgIA4 Potently Blocks Mouse α9α10 nAChRs and Provides Long Lasting Protection against Oxaliplatin-Induced Cold Allodynia. Front. Cell. Neurosci..

[14] Dyachenko I.A., Palikova Y.A., Palikov V.A., Korolkova Y.V., Kazakov V.A., Egorova N.S., Garifulina A.I., Utkin Y.N., Tsetlin V.I., Kryukova E.V. (2022). α-Conotoxin RgIA and oligoarginine R8 in the mice model alleviate long-term oxaliplatin induced neuropathy. Biochimie.

[15] Huynh P.N., Christensen S.B., McIntosh J.M. (2022). RgIA4 Prevention of Acute Oxaliplatin-Induced Cold Allodynia Requires α9-Containing Nicotinic Acetylcholine Receptors and CD3(+) T-Cells. Cells.

[16] Pacini A., Micheli L., Maresca M., Branca J.J., McIntosh J.M., Ghelardini C., Di Cesare Mannelli L. (2016). The α9α10 nicotinic receptor antagonist α-conotoxin RgIA prevents neuropathic pain induced by oxaliplatin treatment. Exp. Neurol..

[17] Romero H.K., Christensen S.B., Di Cesare Mannelli L., Gajewiak J., Ramachandra R., Elmslie K.S., Vetter D.E., Ghelardini C., Iadonato S.P., Mercado J.L. (2017). Inhibition of α9α10 nicotinic acetylcholine receptors prevents chemotherapy-induced neuropathic pain. Proc. Natl. Acad. Sci. USA.

[18] Li X., Hu Y., Wu Y., Huang Y., Yu S., Ding Q., Zhangsun D., Luo S. (2016). Anti-hypersensitive effect of intramuscular administration of αO-conotoxin GeXIVA[1,2] and GeXIVA[1,4] in rats of neuropathic pain. Prog. Neuro-Psychopharmacol. Biol. Psychiatry.

[19] Mohammadi S.A., Burton T.J., Christie M.J. (2017). α9-nAChR knockout mice exhibit dysregulation of stress responses, affect and reward-related behaviour. Behav. Brain Res..

[20] Chang H.H.V., Morley B.J., Cullen K.E. (2021). Loss of α-9 Nicotinic Acetylcholine Receptor Subunit Predominantly Results in Impaired Postural Stability Rather Than Gaze Stability. Front. Cell. Neurosci..

[21] Wala E.P., Crooks P.A., McIntosh J.M., Holtman J.R. (2012). Novel small molecule α9α10 nicotinic receptor antagonist prevents and reverses chemotherapy-evoked neuropathic pain in rats. Anesth. Analg..

[22] Robinson C.R., Dougherty P.M. (2015). Spinal astrocyte gap junction and glutamate transporter expression contributes to a rat model of bortezomib-induced peripheral neuropathy. Neuroscience.

[23] Doyle T., Chen Z., Muscoli C., Bryant L., Esposito E., Cuzzocrea S., Dagostino C., Ryerse J., Rausaria S., Kamadulski A. (2012). Targeting the overproduction of peroxynitrite for the prevention and reversal of paclitaxel-induced neuropathic pain. J. Neurosci. Off. J. Soc. Neurosci..

[24] Qin B., Li Y., Liu X., Gong D., Zheng W. (2020). Notch activation enhances microglial CX3CR1/P38 MAPK pathway in rats model of vincristine-induced peripheral neuropathy. Neurosci. Lett..

[25] Navia-Pelaez J.M., Choi S.H., Dos Santos Aggum Capettini L., Xia Y., Gonen A., Agatisa-Boyle C., Delay L., Gonçalves Dos Santos G., Catroli G.F., Kim J. (2021). Normalization of cholesterol metabolism in spinal microglia alleviates neuropathic pain. J. Exp. Med..

[26] Brandolini L., d’Angelo M., Antonosante A., Allegretti M., Cimini A. (2019). Chemokine Signaling in Chemotherapy-Induced Neuropathic Pain. Int. J. Mol. Sci..

[27] Vichaya E.G., Chiu G.S., Krukowski K., Lacourt T.E., Kavelaars A., Dantzer R., Heijnen C.J., Walker A.K. (2015). Mechanisms of chemotherapy-induced behavioral toxicities. Front. Neurosci..

[28] Lee J.H., Kim W. (2020). The Role of Satellite Glial Cells, Astrocytes, and Microglia in Oxaliplatin-Induced Neuropathic Pain. Biomedicines.

[29] Stephens K.E., Chen Z., Sivanesan E., Raja S.N., Linderoth B., Taverna S.D., Guan Y. (2018). RNA-seq of spinal cord from nerve-injured rats after spinal cord stimulation. Mol. Pain.

[30] Echeverria-Villalobos M., Tortorici V., Brito B.E., Ryskamp D., Uribe A., Weaver T. (2023). The role of neuroinflammation in the transition of acute to chronic pain and the opioid-induced hyperalgesia and tolerance. Front. Pharmacol..

[31] Grau V., Richter K., Hone A.J., McIntosh J.M. (2018). Conopeptides [V11L;V16D]ArIB and RgIA4: Powerful Tools for the Identification of Novel Nicotinic Acetylcholine Receptors in Monocytes. Front. Pharmacol..

[32] Tsetlin V., Haufe Y., Safronova V., Serov D., Shadamarshan P., Son L., Shelukhina I., Kudryavtsev D., Kryukova E., Kasheverov I. (2021). Interaction of α9α10 Nicotinic Receptors With Peptides and Proteins From Animal Venoms. Front. Cell. Neurosci..

[33] AlSharari S.D., Toma W., Mahmood H.M., Michael McIntosh J., Imad Damaj M. (2020). The α9α10 nicotinic acetylcholine receptors antagonist α-conotoxin RgIA reverses colitis signs in murine dextran sodium sulfate model. Eur. J. Pharmacol..

[34] Liu Q., Li M., Whiteaker P., Shi F.D., Morley B.J., Lukas R.J. (2019). Attenuation in Nicotinic Acetylcholine Receptor α9 and α10 Subunit Double Knock-Out Mice of Experimental Autoimmune Encephalomyelitis. Biomolecules.

[35] Zhang X., Lei B., Yuan Y., Zhang L., Hu L., Jin S., Kang B., Liao X., Sun W., Xu F. (2020). Brain control of humoral immune responses amenable to behavioural modulation. Nature.

[36] Richter K., Herz S.M., Stokes C., Damaj M.I., Grau V., Papke R.L. (2023). Pharmacological profiles and anti-inflammatory activity of pCN-diEPP and mCN-diEPP, new α9α10 nicotinic receptor ligands. Neuropharmacology.

[37] Zhu X., Yuan M., Wang H., Zhangsun D., Yu G., Che J., Luo S. (2022). Novel αO-conotoxin GeXIVA[1,2] Nonaddictive Analgesic with Pharmacokinetic Modelling-Based Mechanistic Assessment. Pharmaceutics.

[38] Milligan E.D., Watkins L.R. (2009). Pathological and protective roles of glia in chronic pain. Nat. Rev. Neurosci..

[39] Di Cesare Mannelli L., Pacini A., Micheli L., Tani A., Zanardelli M., Ghelardini C. (2014). Glial role in oxaliplatin-induced neuropathic pain. Exp. Neurol..

[40] Di Cesare Mannelli L., Pacini A., Bonaccini L., Zanardelli M., Mello T., Ghelardini C. (2013). Morphologic features and glial activation in rat oxaliplatin-dependent neuropathic pain. J. Pain.

[41] Di Cesare Mannelli L., Cinci L., Micheli L., Zanardelli M., Pacini A., McIntosh J.M., Ghelardini C. (2014). α-conotoxin RgIA protects against the development of nerve injury-induced chronic pain and prevents both neuronal and glial derangement. Pain.

[42] Janes K., Wahlman C., Little J.W., Doyle T., Tosh D.K., Jacobson K.A., Salvemini D. (2015). Spinal neuroimmune activation is independent of T-cell infiltration and attenuated by A3 adenosine receptor agonists in a model of oxaliplatin-induced peripheral neuropathy. Brain Behav. Immun..

[43] Elgoyhen A.B. (2023). The α9α10 acetylcholine receptor: A non-neuronal nicotinic receptor. Pharmacol. Res..

[44] Zimmermann M. (1983). Ethical guidelines for investigations of experimental pain in conscious animals. Pain.

[45] Dowell C., Olivera B.M., Garrett J.E., Staheli S.T., Watkins M., Kuryatov A., Yoshikami D., Lindstrom J.M., McIntosh J.M. (2003). α-conotoxin PIA is selective for α6 subunit-containing nicotinic acetylcholine receptors. J. Neurosci. Off. J. Soc. Neurosci..

[46] Dixon W.J. (1980). Efficient analysis of experimental observations. Annu. Rev. Pharmacol. Toxicol..

[47] Deuis J.R., Dvorakova L.S., Vetter I. (2017). Methods Used to Evaluate Pain Behaviors in Rodents. Front. Mol. Neurosci..

[48] Li X., Xiong J., Zhang B., Zhangsun D., Luo S. (2021). α-Conotoxin TxIB Inhibits Development of Morphine-Induced Conditioned Place Preference in Mice via Blocking α6β2* Nicotinic Acetylcholine Receptors. Front. Pharmacol..

[49] Love M.I., Huber W., Anders S. (2014). Moderated estimation of fold change and dispersion for RNA-seq data with DESeq2. Genome Biol..

[50] Mao K., Li X., Chen Z., Dong X., Zhangsun D., Zhu X., Luo S. (2022). α-Conotoxin TxIB Improved Behavioral Abnormality and Changed Gene Expression in Zebrafish (Danio rerio) Induced by Alcohol Withdrawal. Front. Pharmacol..

[51] Yu G., Wang L.G., Han Y., He Q.Y. (2012). clusterProfiler: An R package for comparing biological themes among gene clusters. Omics A J. Integr. Biol..

[52] Chen C., Wu Y., Li J., Wang X., Zeng Z., Xu J., Liu Y., Feng J., Chen H., He Y. (2023). TBtools-II: A “one for all, all for one” bioinformatics platform for biological big-data mining. Mol. Plant.

