# Supplementary material for: αO-Conotoxin GeXIVA[1,2] Reduced Neuropathic Pain and Changed Gene Expression in Chronic Oxaliplatin-Induced Neuropathy Mice Model
Source: Mar Drugs. 2024 Jan 19;22(1):49. doi: 10.3390/md22010049 (PMC10821445; doi:10.3390/md22010049)
Supplement: Supplementary file 1 [file marinedrugs-22-00049-s001.zip › Supplementary Figure S1.pdf]

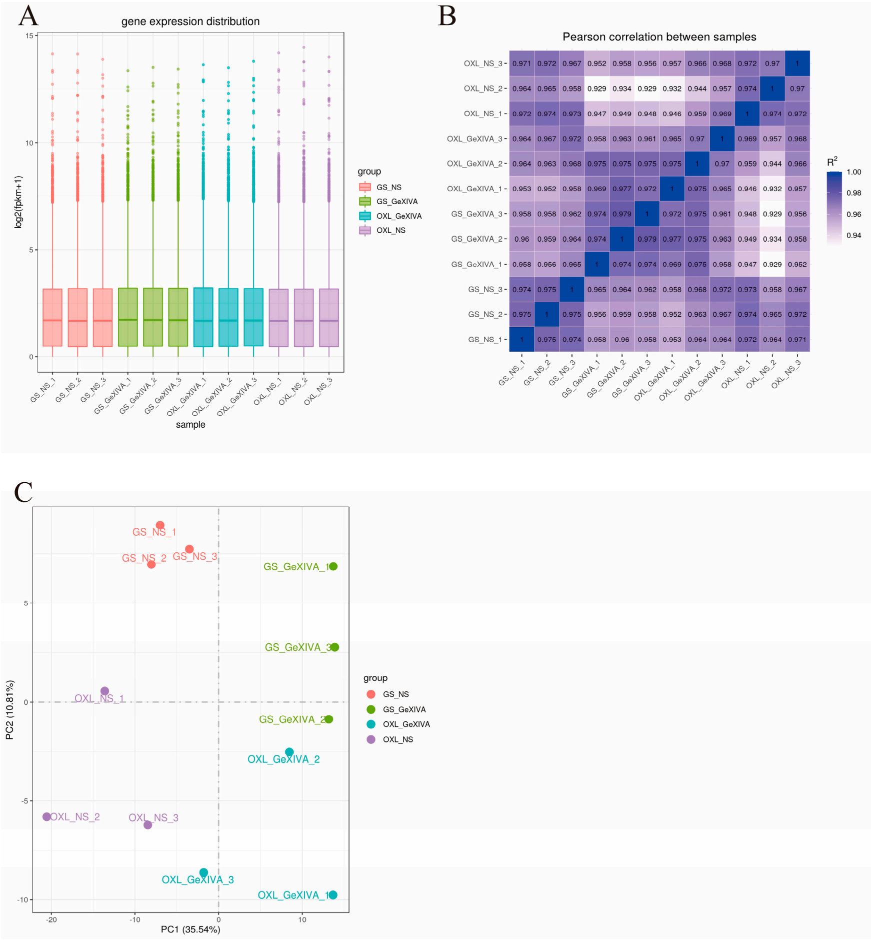

Supplementary Figure S1**.** RNA-sequencing results. (A) The distribution of gene expression levels in each sample. (B) Heatmap of pearson correlation between samples. (C) Principal component analysis of all samples.
